# Effectiveness and safety of electroacupuncture for poststroke patients with shoulder pain: study protocol for a double-center, randomized, patient- and assessor-blinded, sham-controlled, parallel, clinical trial

**DOI:** 10.1186/s12906-019-2468-x

**Published:** 2019-03-12

**Authors:** Seungwon Shin, Sung Pil Yang, Ami Yu, Junghee Yoo, Sung Min Lim, Euiju Lee

**Affiliations:** 1 Department of Clinical Korean Medicine, Graduate School, Kyung Hee University, 26 Kyungheedae-ro, Dongdaemun-gu, Seoul, 02447 Republic of Korea; 2 Korean Medicine Clinical Trial Center, Kyung Hee University Korean Medicine Hospital, 23 Kyungheedae-ro, Dongdaemun-gu, Seoul, 02447 Republic of Korea; 3 College of Nursing Science, Kyung Hee University, 23 Kyungheedae-ro, Dongdaemun-gu, Seoul, 02447 Republic of Korea; 4 Department of Clinical Research on Rehabilitation, Korea National Rehabilitation Research Institute, 58 Samgaksan-ro, Gangbuk-gu, Seoul, 01022 Republic of Korea; 5 College of Korean Medicine, Kyung Hee University, 23 Kyungheedae-ro, Dongdaemun-gu, Seoul, 02447 Republic of Korea

**Keywords:** Stroke, Shoulder pain, Electroacupuncture, Randomized controlled trial

## Abstract

**Background:**

Practitioners of complementary and alternative medicine have suggested that acupuncture could alleviate poststroke shoulder pain, based on the clinical evidence. This study protocol is aimed at showing the effectiveness and safety of electroacupuncture therapy for stroke survivors with shoulder pain.

**Methods:**

After assessing their eligibility, 60 stroke survivors with shoulder pain will be enrolled from two traditional Korean medicine hospitals and randomly divided into either the verum or the sham electroacupuncture (EA) group with a 1:1 ratio. The participants will receive 9 sessions of EA procedures for 3 weeks. The verum EA consists of needling on 6 unilateral acupoints (LI4, LI15, TE14, SI9, SI11, and GB21) with electronic stimulation. A non-penetrating Park sham device and fake electronic stimulation will be used in the sham group on the same acupoints. Patients and outcome assessors will be blinded throughout the entire study. A visual analog scale will be used primarily for the evaluation, and pain rating scale, Fugl-Meyer assessment for upper extremity, modified Ashworth scale, manual muscle test, passive range of motion test, Korean version of a modified Barthel index, and Korean version of the Beck depression inventory will be also be measured. A blinding index will be assessed. For safety, adverse events will be recorded. Data will be statistically analyzed by two-sample t-test or Wilcoxon rank sum test for efficacy and a chi-squared test or Fisher’s exact test for safety, at 5% of significance level.

**Discussion:**

We expect this double-center, randomized, sham-controlled, patient- and assessor-blinded parallel trial to explore the effectiveness and safety of EA therapy, compared with sham EA, for poststroke shoulder pain.

**Trial registration:**

https://clinicaltrials.gov/ct2/show/NCT03086863

## Background

Hemiplegic shoulder pain (HSP) is a common complication of stroke survivors. It is estimated that HSP occurs in 16 to 84% of stroke patients [1] or 22 to 23% of the general population [2]. Due to HSP, poststroke patients may have difficulty with activities of rehabilitation and a deteriorated quality of life [3], which is why management or treatment of HSP is considered important.

Various conditions, such as structural injury from glenohumeral subluxation, capsular contractures, or rotator cuff disorders, are known to cause the pain [3]. It is specifically stated that impingement syndrome, brachial plexus injury, frozen shoulder, poststroke central pain, shoulder hand syndrome, myofascial pain syndrome, tendinitis, or bursitis could be related to the poststroke shoulder pain [4]; therefore, several curative methods have been applied and studied.

An evidence-based clinical practice guideline for stroke rehabilitation in Korea recommended shoulder support systems, physiotherapy, analgesics, intraarticular steroid injection, or botulinum toxin A (BTX-A) [5]. However, systematic reviews have discussed the pros and cons of intramuscular neuromuscular electric stimulation [3, 6], steroid injection [3, 6], BTX-A injection [3, 6, 7], shoulder supports [3], and even analgesia [3], which suggests that the treatments for HSP are controversial and inconsistent.

Practitioners in the field of complementary and alternative medicine suggest that acupuncture therapy can alleviate poststroke shoulder pain according to the evidence from systematic reviews and meta-analyses [8–10]. However, it fell short of the adequate randomized controlled trials (RCTs) and did not have the study quality or numbers to prove the effect of electroacupuncture (EA) therapy for HSP [10].

The objective of this study protocol is to conduct a double-center, randomized, patient- and assessor-blinded, sham-controlled, parallel clinical trial in order to show the effectiveness and safety of EA therapy for stroke survivors with HSP.

## Methods/design

### Aim, design, and setting of the study

#### Primary objective and study hypothesis

This clinical trial is primarily aimed at evaluating the effectiveness and safety of 3-week EA therapies, compared to sham comparator, for patients with shoulder pain that is rated on the visual analog scale (VAS) as ≥4 after a stroke, such as an intracerebral hemorrhage or infarction. The alternative hypothesis is that the mean difference in VAS for HSP, from baseline to endpoint, of the verum group is not equal to that of the sham group.

#### Secondary objectives

This study will also evaluate the effects of EA on a) physical performance, spasticity, muscle strength, and range of motion in shoulder joints, b) activities of daily living, and c) depression of stroke survivors, using intergroup/intragroup comparisons of various validated scales.

#### Study design

This clinical study is a double-center, randomized, sham-controlled, patient- and assessor-blinded, and parallel trial. A total of 60 stroke survivors will be given both verbal and written forms of in-depth information about the trial by traditional Korean medicine (TKM) doctors before participation. All participants will voluntarily sign the informed consent that has been approved by the ethics committee prior to enrollment. Prior to allocation, the enrolled participants will be screened by the eligibility criteria.

After being randomly allocated into either the verum or sham EA group, in a 1:1 of allocation ratio, the participant will receive 9 sessions of verum or sham EA procedures for 3 weeks (3 sessions a week). The acupuncture regimen consists of 6 unilateral acupoints (LI4, LI15, TE14, SI9, SI11, and GB21) with electronic stimulation. Patients and outcome assessors will be blinded from the beginning of the study through completion.

**Fig. 1:**
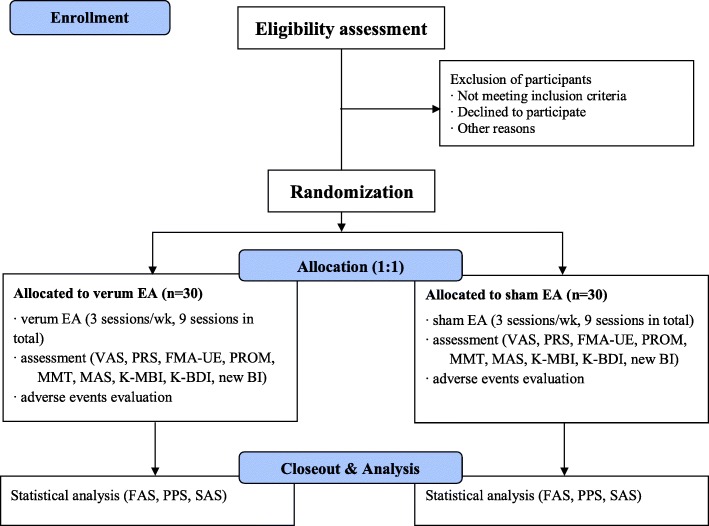
Schematic chart of the overall study. The template is from the CONSORT 2010 flow diagram. *EA* electroacupuncture, *VAS* visual analog scale, *PRS* pain rating scale, *FMA-UE* Fugl-Meyer assessment upper extremity, *PROM* passive range of motion, *MMT* manual muscle test, *MAS* modified Ashworth scale, *K-MBI* Korean version of modified Barthel index, *K-BDI* Korean version of Beck depression inventory, *BI* blinding index, *FAS* full analysis set, *PPS* per protocol analysis set, *SAS* safety assessment set

**Fig. 2:**
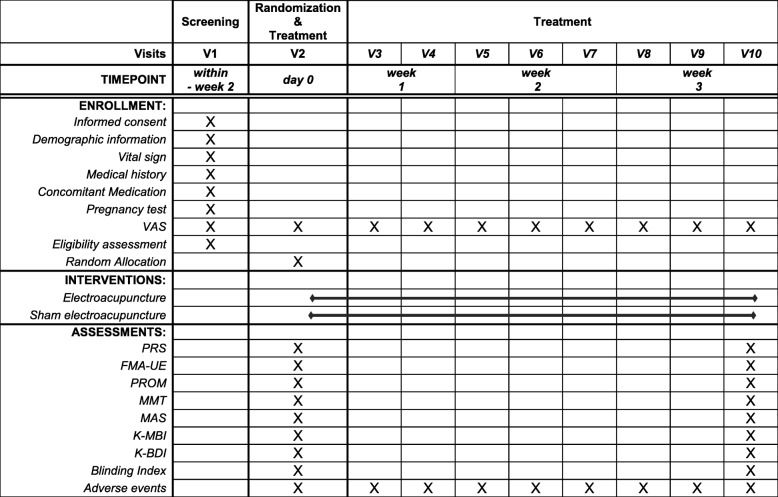
Study schedule of enrollment, interventions, and assessments. The template is from the SPIRIT 2013 statement [11]. *VAS* visual analog scale, *PRS* pain rating scale, *FMA-UE* Fugl-Meyer assessment upper extremity, *PROM* passive range of motion, *MMT* manual muscle test, *MAS* modified Ashworth scale, *K-MBI* Korean version of modified Barthel index, *K-BDI* Korean version of Beck depression inventory

The overall schematic chart and study schedule are shown in Figs. 1 and 2, respectively.

#### Setting and recruitment

Two TKM hospitals (Kyunghee University Korean Medicine Hospital, Seoul; Wonkwang University Gwangju Medical Center, Gwangju) in the Republic of Korea will recruit eligible participants. Both hospitals will post study advertisements online (webpage) and offline (posters) to enroll inpatients. To enhance recruitment, investigators will also monitor feasible medical records.

#### Ethical review and general statements

This trial protocol (version 1.5), including the informed consent forms, has been peer-reviewed and approved by the funder and the Institutional Review Board at Kyung Hee University Korean Medicine Hospital (approval No. KOMCIRB-170215-HR-002) according to the scientific content and ethical compliance with regulations, i.e., Good Clinical Practice and relevant laws by Ministry of Food and Drug Safety in Korea. The study has also been registered at ClinicalTrials.gov in March 2017 (identification No. NCT03086863).

We designed this interventional research at full length following the Standard Protocol Items: Recommendations for Interventional Trials (SPIRIT) 2013 statement [11] and Revised Standards for Reporting Interventions in Clinical Trials of Acupuncture (STRICTA): extending the CONSORT statement [12, 13].

### Eligibility criteria

#### Inclusion criteria

We will include any man or woman who a) is 19 years of age or older; b) was diagnosed with cerebral hemorrhage or infarction through an examination using computed tomography (CT) magnetic resonance imaging (MRI) at least 2 weeks prior to the enrollment; c) has hemiplegic shoulder pain with a VAS score ≥ 4; d) agrees that any treatments, including analgesics, for HSP will not be changed from the previous 2 weeks prior to the enrollment until the last evaluation, if applicable; and e) has received a full explanation about the research and has given informed consent.

#### Exclusion criteria

Participants will be excluded if they have/had a) disorders, traumatic injury, or surgery of shoulders even before their stroke; b) pacemakers, embedded neural stimulator, cardiac arrhythmia, epilepsy, peripheral neural injury on their medical history; c) psychiatric disorders; d) cancer within the past 5 years, regardless of its prognosis and location; e) cognitive impairment that interferes with clinical assessment; f) hypersensitivity to or fear of acupuncture; g) bleeding disorders (e.g. hemophilia or von Willebrand disease, etc.), h) a pregnancy, i) difficulty in communicating with researchers, or j) any other conditions that are considered inappropriate for participating in the trial by experienced practitioners.

#### Dropout criteria

Any participant will be dropped from the study if he or she a) has a belated discovery of any ineligible criteria; b) withdraws consent, wants to cease, or refuses to undergo the EA or sham procedures; c) is lost to follow-up; d) shows serious adverse events and fails to maintain the protocol; e) takes concomitant use of the prohibited drugs or therapies (see the section, Concomitant treatments); f) violates the protocol seriously; or g) is considered inappropriate to keep in the trial by investigators’ clinical experience.

### Randomization and allocation

Random numbers will be generated by an independent statistician (A. Yu) using SAS® 9.4 software (SAS Institute Inc., North Carolina, USA). Block randomization with randomly selected block sizes of either 2 or 4 will also be adopted to keep the number of participants in each group as equal as possible at all times with an allocation ratio of 1:1. Of the 60 total participants, 40 will be stratified by site subgroups (20 in Kyunghee University Korean Medicine Hospital and 20 in Wonkwang University Gwangju Medical Center, respectively) and the remaining 20 will be competitively recruited from both sites.

Sealed and opaque envelopes with random numbers will be prepared by the statistician and opened by a study coordinator after eligibility assessment. Participants will be allocated into either the verum or sham EA group in the order of giving the informed consents. The study coordinator will notify the allocated group to practitioners, who will be different from outcome assessors.

### Blinding

We will use the non-penetrating Park-sham apparatus as a comparator. The same guide tube from the Park-sham needle will be used in both groups and, simultaneously, a fake noise from an identical electrical stimulator will be generated to maintain blinding during the procedures (see the section, Sham electroacupuncture). In addition, outcome assessors will be blinded, who are different from the study coordinators, TKM doctors performing the acupuncture procedures, and statistician. Unblinding before completing the study will only be allowed in cases of medical emergencies induced by serious adverse events (SAEs). At the end of the study, patients and outcome assessors, respectively, should try to deduce which group they thought to belong to. These answers will be used to calculate the new blinding index (BI) to evaluate success or failure of blinding [14].

### Verum electroacupuncture

The detailed procedures of EA are described following the STRICTA 2010 checklist [12, 13].

The regimen of EA therapy is composed based on the TKM’s theory on acupuncture and the clinical experiences of the TKM experts. All the patients in the verum EA group will go through an identical procedure. On the six adjacent acupoints (LI4, LI15, TE14, SI9, SI11, and GB21), placed on only the side with HSP, the acupuncture needles (stainless steel, 0.25 mm × 40 mm, Dong Bang Acupuncture Inc., Republic of Korea) will be inserted at a depth of 10–15 mm and the de qi sensation will be elicited. Park sham guide tubes will be also used in the verum EA group to blind the patients. The locations of each acupoint conforms to the standard defined by the World Health Organization [15]. Subsequently, a low frequency electronical stimulate (STN-111, Stratek, Republic of Korea) will be connected to the tips of needles to provide electrical stimulation at a medium frequency (30 Hz). Needles with electrical stimulation will be retained for 20 min.

Patients should undergo this procedure 3 times a week for 3 weeks (9 sessions in total). During the procedures, the practitioners are not allowed to talk to patients, minimizing other effects. The practitioners will be licensed TKM doctors with at least 1 year of clinical experience. These practitioners cannot assess the outcomes because assessors should remain blinded throughout the whole study.

### Sham electroacupuncture

This study has been designed to show the effectiveness and safety of EA, compared to a sham control, on HSP. Therefore, we selected non-penetrating sham needling (Park sham device), which has been developed and validated by previous studies [16, 17] for comparison. The needles will be placed using the Park sham guide tubes exactly at the same 6 acupoints as the verum group and the same electrical stimulators will be connected to the tips of the needles. Since the needles do not penetrate the skin, the stimulation is not electrically conducted. To ensure patient blinded, the low frequency stimulate will be turned on with the same frequency (30 Hz), which makes a fake noise, resulting in the patients believing that they are receiving the real EA therapy for 20 min. The sham group will also complete 9 sessions over a period of 3 weeks (3 sessions a week).

### Concomitant treatments

Drugs, such as steroids or nonsteroidal anti-inflammatory drugs, herbal medications, acupuncture, moxibustion, or rehabilitation therapies for shoulder pain in stroke patients are allowed throughout the trial. However, all concomitant treatments for HSP should be maintained with the same dosage and regimen at least 2 weeks before enrollment until the last assessment. If necessary, the participant should take 2 weeks for a run-in period before enrollment. When there are significant changes of concomitant medications in an enrolled participant, he or she will be dropped out.

The typical care for cerebral hemorrhage or infarction, including oral medications (antiplatelet agents, anticoagulants, or neuroprotectants), rehabilitation therapies, traditional herbal medications, acupuncture, moxibustion, and EA therapies except for at the LI4, LI15, TE14, SI9, SI11, and GB21 acupoints will be permitted throughout the study for both groups.

All the treatments that the enrolled patients take will be monitored closely and recorded in case report forms. When any concomitant treatments related to shoulder pain are significantly different between groups, the findings will statistically be adjusted with these data of concomitant treatments.

### Outcomes

#### Primary outcome

The VAS is a patient-rated outcome which has been validated for pain [18]. This scale is a 10-cm line where zero represents “not painful at all” and ten represents “most painful.” The patient marks an “x” on the line, based on how intense he/she thinks the pain is, and the assessor measures the length from zero to the “x” mark, which is the pain score. Many clinical trials for poststroke shoulder pain use VAS as their main outcomes [10]. In this study, VAS will be evaluated at every visit and the primary endpoint is defined as the mean change of VAS between the baseline and endpoint to show the intergroup difference. The mean differences of the before and after VAS scores within each group will be subordinately evaluated to see the intragroup effect.

#### Secondary outcomes

The pain rating scale (PRS) is also going to be measured for shoulder pain. PRS is another simple and comprehensive patient-rated outcome used to assess pain. It consists of 4 items for intensity (0–10 points), frequency (0–5 points), duration (0–5 points), and aggravating factors (0–5 points). The pain score is the product of the intensity point and the sum of frequency, duration, and aggravating factors points (0–150 points). The reliability and validity of PRS has been evaluated in a previous study [19].

The Fugl-Meyer assessment is a validated tool that assesses physical performance following a stroke [20]. The scale includes the 4 domains of motor function, quality of sensation, passive range of motion, and joint pain, which are assessed using a 3-point scale (0: “cannot perform”; 1: “perform partially”; and 2: “perform fully”). We plan to only measure 8 items for the upper extremity, (Fugl-Meyer assessment of the upper extremity, FMA-UE), i.e., shoulder retraction, elevation, abduction, abduction to 90°, adduction/internal rotation, external rotation, flexion 0–90°, and flexion 90–180°, which follows the precedent [21].

Passive ranges of motion (PROM) for shoulder flexion, abduction, and extension will be measured with goniometer. We will test the maximum angle of passive movement of the shoulder, unless patients suffer from pain.

A manual muscle test (MMT) will also be included to assess muscle strength with a poststroke shoulder injury. Assessors will grade the hemiplegic shoulder on a scale of 0 (no contraction at all) to 5 (complete range of motion against gravity with maximum resistance) [22, 23].

The modified Ashworth scale (MAS) is one of the most frequently adapted scales for poststroke spasticity. The scale was developed and validated as a clinical rating scale to measure tonal abnormality [24]. Assessors should grade from 0 (no increase in muscle tone) to 5 (rigid shoulder on flexion or extension).

The Korean version of the modified Barthel index (K-MBI) is known to be an ordinal scale used to measure performance in activities of daily living [25]. Ten items (personal hygiene, bathing, feeding, toilet use, stair climbing, dressing, bowel control, bladder control, walking, and chair/bed transfers) will be graded on scale of 1 (completely dependent on others to perform) to 5 (completely independent on others to perform), with a maximum total score of 100 points. The reliability and validity of the Korean version of this scale have been studied [25].

The Korean version of the Beck depression inventory (K-BDI) will be also included in the outcome measurement, on the grounds that depression is prevalent in stroke patients and it could have a negative effect on their functional outcomes [26]. This is a patient-rated outcome with 21 items, 0 to 3 points for each item. A higher the total score reflects a more severe level of depression. This scale has also been validated in Korean [27, 28].

Outcome assessors will be licensed TKM doctors who are not involved in the allocation or EA procedures. They are qualified only when they have greater than 1 year of clinical experience. All the secondary outcomes will be evaluated at baseline and at the end of the study. The mean differences between the verum and sham groups will be compared.

#### Blinding index

Patient and assessor blinding will be evaluated separately with a new BI [14]. They are going to be asked which group they think they belong to and select one of the following answers: verum EA group, sham EA group, or unknown. The index score varies from 1 (complete lack of blinding), 0 (consistent with perfect blinding), or − 1 (guessed they were in the opposite group). This will be assessed at the end of study.

#### Safety assessment

At every visit, assessors will ask a post-interventional question pertaining to adverse events. Unpleasant feelings regarding needling, dizziness, allergic reactions, slight pain, bruises, or pinpoint bleeding are expected on an individual basis. The intensity and causality of each adverse event will be evaluated and recorded. If serious adverse events occur, post-management will be properly carried out. The occurrences number of adverse events will be used for statistical analysis.

### Power calculation

The primary endpoint is the mean difference of the VAS scores for poststroke shoulder pain between the baseline and the endpoint. The null hypothesis is that μ_v_ = μ_s_*,* while the alternative hypothesis is *μ*_*v*_ ≠ *μ*_*s*_, where *μ* is the mean difference and *v* and *s* denotes the verum or sham EA group, respectively. A previous study to compare the effectiveness of acupuncture therapy using non-acupoint sham acupuncture on poststroke shoulder pain [29] showed the reductions of VAS scores with means ± standard deviations of 3.52 ± 2.67 in the intervention group and 1.38 ± 2.43 in control group, respectively. Using a two-sided test, under the assumptions of an of 0.05 and (1-β) of 0.8, a sample size of 24 per group has been calculated. Considering a 20% of dropout rate, a total of 60 participants will be enrolled in the trial (30 per group).

### Statistical analysis

A full analysis set is defined as a group of participants who receive 7 or more verum or sham EA sessions with an evaluation of the primary outcome at the endpoint, while a per protocol set is a subset of participants who complete all verum or sham EA sessions as planned in the protocol. We are going to statistically analyze the obtained data, primarily on the full analysis set, as well as, on the per protocol set for the efficacy analysis. Missing values will be imputed by the method of last observation carried forward in the full analysis set.

Demographic information will be tested with a two-sample t-test or Wilcoxon rank sum test for continuous variables and chi-squared test or Fisher’s exact test for categorical variables. When statistically significant differences between groups are found in demographic information, especially age and sex, those will be used as covariates for effectiveness analysis.

Primary and secondary variables will be summarized using the mean ± standard deviation and tested by two-sample t-test or Wilcoxon rank sum test depending on normality of the distribution. If concomitant treatments are significantly different between groups, intergroup effect will be tested by an analysis of covariance with the information of concomitant treatments adjusted. For the intragroup effect, the results of the VAS scores will be tested by paired t-test or Wilcoxon signed rank test. The significance level will be set at 0.05 (two-sided). Statistical analyses will be performed using SAS® 9.4 software (SAS Institute Inc., North Carolina, USA) by an independent statistician (A. Yu).

The number of adverse events will be presented with a 95% confidence interval on a safety set defined as a subset of participants who receive at least one verum or sham treatment. A chi-squared test or Fisher’s exact test will be carried out to compare the significant differences between groups.

The results of the blinding assessment will be presented as the number of participants per group assignment with the participants’ deductions of their group assignment in a 2 × 3 table. We will consider blinding to be successful in this study if the calculated confidence interval of the new BI includes zero, following the previous study [14].

## Discussion

This is the study protocol of a double-center, randomized, sham-controlled, patient- and assessor-blinded parallel trial to explore the effectiveness and safety of EA therapy, compared to a sham EA therapy, for poststroke shoulder pain. The eligible patients will be recruited in 2 TKM hospitals in the Republic of Korea.

Acupuncture therapies have been clinically studied, and based on the evidence from those studies, clinical practice guidelines have been published for general rehabilitation [30] or specific conditions of stroke survivors [31, 32]. In a systematic review, acupuncture combined with rehabilitation therapy showed therapeutic effect for shoulder pain occurring after a stroke [9]; however, clinical evidence for EA therapy for the same condition is generally lacking [9, 10]. For this reason, we have chosen to use EA intervention combined with the usual therapy for poststroke shoulder pain. In addition, previous EA studies do not compare the intervention with validated sham devices [33, 34], which made us consider the necessity of a sham-controlled trial.

There is no clear evidence for the most suitable time to begin treating shoulder pain after a stroke occurs. However, approximately 72% of individuals with HSP are estimated to develop symptoms 2 weeks after the onset of stroke [3]. We will recruit patients who are in this poststroke phase to show overall effectiveness.

A literature review addressed the selected acupoints (LI4, LI15, TE14, SI9, SI11, and GB21) that have been proposed to treat general shoulder pain, which are based on the standards of TKM [35]. Additionally, most acupoints are anatomically located around the muscles involving shoulder movement. Therefore, we expect EA treatment on these acupoints to be beneficial for poststroke shoulder pain, similar to the results presented in a systematic review on the stimulation of Ashi points for general shoulder pain [36].

## Abbreviations

BI: blinding index
BTX-A: botulinum toxin A
CT: computed tomography
EA: electroacupuncture
FMA-UE: Fugl-Meyer assessment upper extremity
HSP: hemiplegic shoulder pain
K-BDI: Korean version of Beck depression inventory
K-MBI: Korean version of modified Barthel index
MAS: modified Ashworth scale
MMT: manual muscle test
MRI: magnetic resonance imaging
PROM: passive range of motion
PRS: pain rating scale
RCT: randomized controlled trial
SAE: serious adverse event
SPIRIT: standard protocol items: recommendations for interventional trials
STRICTA: revised standards for reporting interventions in clinical trials of acupuncture
TKM: traditional Korean medicine
VAS: visual analog scale
